# Do the negative symptoms of schizophrenia reflect reduced responsiveness to reward? Examination using a reward prediction error (RPE) task

**DOI:** 10.1017/S0033291723000521

**Published:** 2023-11

**Authors:** Paola Fuentes-Claramonte, Maria Angeles Garcia-Leon, Pilar Salgado-Pineda, Núria Ramiro, Joan Soler-Vidal, Maria Llanos Torres, Ramon Cano, Isabel Argila-Plaza, Francesco Panicali, Carmen Sarri, Núria Jaurrieta, Manel Sánchez, Ester Boix-Quintana, Auria Albacete, Teresa Maristany, Salvador Sarró, Joaquim Raduà, Peter J. McKenna, Raymond Salvador, Edith Pomarol-Clotet

**Affiliations:** 1 FIDMAG Germanes Hospitalàries Research Foundation, Barcelona, Spain; 2 CIBERSAM (Centro de Investigación Biomédica en Red de Salud Mental), Instituto de Salud Carlos III, Barcelona, Spain; 3 Hospital Sant Rafael, Barcelona, Spain; 4 Benito Menni CASM, Sant Boi de Llobregat, Barcelona, Spain; 5 Universitat de Barcelona, Barcelona, Spain; 6 Hospital Mare de Déu de la Mercè, Barcelona, Spain; 7 Hospital Sagrat Cor, Martorell, Barcelona, Spain; 8 Universitat Autònoma de Barcelona, Barcelona, Spain; 9 Mental Health Department, Hospital de Mataró, Mataró, Spain; 10 Diagnostic Imaging Department, Hospital Sant Joan de Déu, Barcelona, Spain; 11 Institut d'Investigacions Biomèdiques August Pi i Sunyer (IDIBAPS), Barcelona, Spain; 12 Department of Clinical Neuroscience, Centre for Psychiatric Research and Education, Karolinska Institute, Stockholm, Sweden; 13 Department of Psychosis Studies, Institute of Psychiatry, Psychology and Neuroscience, King's College London, London, UK

**Keywords:** fMRI, negative symptoms, psychosis, reinforcement learning, reward prediction error, reward, schizophrenia

## Abstract

**Background:**

A leading theory of the negative symptoms of schizophrenia is that they reflect reduced responsiveness to rewarding stimuli. This proposal has been linked to abnormal (reduced) dopamine function in the disorder, because phasic release of dopamine is known to code for reward prediction error (RPE). Nevertheless, few functional imaging studies have examined if patients with negative symptoms show reduced RPE-associated activations.

**Methods:**

Matched groups of DSM-5 schizophrenia patients with high negative symptom scores (HNS, *N* = 27) or absent negative symptoms (ANS, *N* = 27) and healthy controls (HC, *N* = 30) underwent fMRI scanning while they performed a probabilistic monetary reward task designed to generate a measure of RPE.

**Results:**

In the HC, whole-brain analysis revealed that RPE was positively associated with activation in the ventral striatum, the putamen, and areas of the lateral prefrontal cortex and orbitofrontal cortex, among other regions. Group comparison revealed no activation differences between the healthy controls and the ANS patients. However, compared to the ANS patients, the HNS patients showed regions of significantly reduced activation in the left ventrolateral and dorsolateral prefrontal cortex, and in the right lingual and fusiform gyrus. HNS and ANS patients showed no activation differences in ventral striatal or midbrain regions-of-interest (ROIs), but the HNS patients showed reduced activation in a left orbitofrontal cortex ROI.

**Conclusions:**

The findings do not suggest that a generalized reduction of RPE signalling underlies negative symptoms. Instead, they point to a more circumscribed dysfunction in the lateral frontal and possibly the orbitofrontal cortex.

Although they make an important contribution to the burden of schizophrenia (e.g. Fervaha, Foussias, Agid, & Remington, 2014; Kirkpatrick, 2014), the negative symptoms of the disorder – lack of volition, poverty of speech and flattening of affect – remain poorly understood. One of two major current approaches is that they are due to impaired prefrontal cortex function. Stemming ultimately from the resemblance between schizophrenic lack of volition and the apathy of neurological patients with the frontal lobe syndrome (Frith, 1992; Liddle, 1987; Weinberger, 1988), this theory is supported principally by neuropsychological studies, which have found significant associations between negative symptom scores and poor performance on a range of executive tests (de Gracia Dominguez, Viechtbauer, Simons, van Os, & Krabbendam, 2009; Dibben, Rice, Laws, & McKenna, 2009), although it should be noted that similar associations have been also found with non-executive tests (de Gracia Dominguez et al., 2009).

The other main explanatory proposal for negative symptoms is that they are due to reduced responsiveness to rewarding stimuli in the environment (for a review see Deserno, Boehme, Heinz, & Schlagenhauf, 2013). This theory grew out of the dopamine hypothesis of schizophrenia, in particular the so-called revised or extended version of this (Davis, Kahn, Ko, & Davidson, 1991; Howes & Kapur, 2009), which proposes that negative symptoms reflect a functional deficiency of this transmitter. It became the subject of significantly increased interest after Schultz, Dayan, and Montague (1997) demonstrated conclusively that phasic release of dopamine from midbrain neurons acts as a reward prediction error (RPE) signal in animals. In humans, functional imaging studies have mapped a series of brain regions that activate in response to RPE (Garrison, Erdeniz, & Done, 2013), as well as to other aspects of reward processing, such as reward anticipation (i.e. when stimuli are viewed that are predictive of reward) and reward feedback (i.e. when information that reward has been won is provided) (Diekhof, Kaps, Falkai, & Gruber, 2012; Jauhar et al., 2021; Liu, Hairston, Schrier, & Fan, 2011; Oldham et al., 2018). Prominent among these regions are the striatum, especially its ventral sector, and the orbitofrontal and adjacent ventromedial frontal cortex, as well as parts of the anterior cingulate cortex and the midbrain.

In schizophrenia, a substantial number of fMRI studies have found that patients show reduced ventral striatal activation during reward anticipation (for meta-analyses see Radua et al., 2015; Zeng et al., 2022). Whether this is also the case outside the ventral striatum is currently the subject of conflicting findings (for meta-analyses see Chase et al., 2018, Leroy et al., 2020; Zeng et al., 2022). At the stage of receiving feedback about reward, findings point to a mixed pattern, with increased activation in schizophreniain the striatum, the amygdala and the hippocampus, plus various cortical regions, coupled with reduced activation in the medial frontal cortex and the dorsolateral frontal cortex (Zeng et al., 2022). The finding of increased activation at this stage is of interest because the RPE signal is generated when feedback about reward gain or loss is given. RPE itself, however, has not been found to be altered in schizophrenia according to a recent meta-analysis (Yaple, Tolomeo, & Yu, 2021), although at 10 the number of studies is so far relatively small.

Although RPE is the aspect of reward processing most closely associated with dopamine, and hence with the ‘reduced responsiveness to reward’ theory of negative symptoms, only a few studies to date have empirically examined the relationship between RPE and these symptoms. Katthagen, Kaminski, Heinz, Buchert, and Schlagenhauf (2020) found that RPE-associated activation within a right ventral striatal region of interest (ROI) was inversely correlated with negative symptoms in 19 patients with schizophrenia. Culbreth, Westbrook, Xu, Barch, and Waltz (2016) found a significant inverse correlation between negative symptoms and RPE-associated activations in a ventral striatal ROI in one of two patient samples (*N* = 28) but not in the other (*N* = 30). Finally, in 38 patients, Dowd, Frank, Collins, Gold, and Barch (2016) found that RPE-related activations were not associated with either clinician- or self-rated anhedonia in an ROI in the striatum.

The aim of the present study was to further investigate the reduced responsiveness to reward theory of negative symptoms. We focused on RPE, on the basis that this is the aspect of reward processing that is most clearly linked to dopamine function. Because correlational analysis has been argued to have limited power to detect brain:behaviour correlations in fMRI studies (Yarkoni, 2009), we used a design comparing groups of schizophrenia patients with and without negative symptoms. A matched healthy control group was also examined.

## Methods

### Participants

The patient sample consisted of two groups of right-handed patients meeting criteria for either high or absent negative symptoms (HNS and ANS, see below). The patients were selected from a larger group of patients recruited from four hospitals in the Barcelona area (Benito Menni CASM, Hospital de Sant Rafael, Hospital Sagrat Cor de Martorell, Hospital Mare de Déu de la Mercè). All patients had a DSM-5 diagnosis of schizophrenia or schizoaffective disorder, made on the basis of clinical interview and review of casenotes.

Patients were excluded if they (a) were younger than 18 or older than 65 years, (b) had a history of brain trauma or neurological disease, or (c) had shown alcohol/substance abuse within 12 months prior to participation. They were also required to have a premorbid IQ in the normal range (⩾70), as estimated using the Word Accentuation Test (Test de Acentuación de Palabras, TAP) (Del Ser, Gonzalez-Montalvo, Martinez-Espinosa, Delgado-Villapalos, & Bermejo, 1997; Gomar et al., 2011), which requires pronunciation of low-frequency Spanish words whose accents have been removed. Patients with a current IQ < 70 based on four subtests from the WAIS-III (Vocabulary, Similarities, Matrix Reasoning and Block Design) were also excluded. All patients were taking antipsychotic medication.

Healthy controls (HC) were recruited from non-medical hospital staff, their relatives and acquaintances, plus independent sources in the community. The HC met the same exclusion criteria as the patients. They were also excluded if they reported a history of mental illness and/or treatment with psychotropic medication, or a history of major mental illness in a first-degree relative. The Structured Clinical Interview for DSM-IV (First, Spitzer, Gibbon, & Williams, 2002) (modified to make it compatible with DSM-5) was administered to exclude current or past psychiatric disorders.

All participants gave written informed consent prior to participation. The study was approved by the ethics committee for the relevant hospitals (Comité d’Ètica i Investigació Clínica de Germanes Hospitalàries). Healthy controls received a gift card as a compensation for their participation.

### Clinical assessment

Negative symptoms were rated using the Positive and Negative Syndrome Scale (PANSS). On the basis of a review of factor analytic studies by Wallwork, Fortgang, Hashimoto, Weinberger, and Dickinson (2012) the following items were considered negative symptoms: blunted affect (N1), emotional withdrawal (N2), poor rapport (N3), apathetic social withdrawal (N4), lack of spontaneity (N6), and motor retardation (G7). All these items are scored in a scale ranging from 1 (absent) to 7 (extreme), indicating the severity of the symptom. Negative symptoms were additionally assessed with the Clinical Assessment Interview for Negative Symptoms (CAINS) (Kring, Gur, Blanchard, Horan, & Reise, 2013; Valiente-Gomez et al., 2015). Scores on individual CAINS items are summed to give an overall score, and also yield two subscale scores: motivation and pleasure (CAINS-MAP, 9 items) focuses on lack of motivation and anhedonia, whereas expressivity (CAINS-EXP, 4 items) rates lack of facial expression and expressive gestures, and prosody and amount of speech.

Patients were assigned to high negative symptoms (HNS) and absent negative symptoms (ANS) groups based on PANSS negative symptom scores, according to criteria devised by Bucci and Galderisi (2017). Patients in the HNS group had scores of at least moderate (i.e. score of 4 out of a maximum of 7) on three or more PANSS negative symptom items, or moderately severe (i.e. 5 or higher) on two or more items. In contrast, patients in the ANS group had scores of either absent or minimal (i.e. a maximum of 2) on all PANSS negative symptom items.

### Task description

**Fig. 1.**
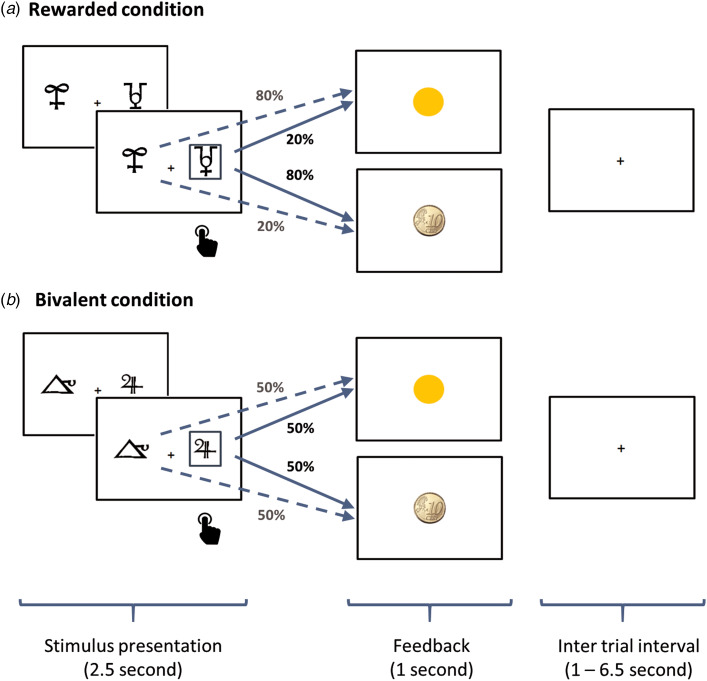
The probabilistic reinforcement learning task. The participant was required to choose through button press one of the two abstract stimuli shown on the screen for 2.5s. The selected stimulus was highlighted and feedback indicating gain (10-cent coin) or no gain (yellow circle) appeared (1s). The participant had to learn by trial and error which of the two stimuli was more likely to yield a reward in order to win as much money as possible. In half of the trials, one of the stimuli had a reward probability of 80% and the other of 20% (rewarded condition). In the other half, both stimuli had the same probability (bivalent condition).

During fMRI scanning, subjects performed a probabilistic monetary learning task adapted from Pessiglione, Seymour, Flandin, Dolan, and Frith (2006). In each trial, two stimuli were shown (one at each side of the screen) for 2.5s. The participant had to select one of them by pressing a button on the left or the right. The selected stimulus was then highlighted and feedback was displayed in the form of either a 10-eurocent coin (gain) or a yellow circle (no gain) (see Fig. 1). After a further 1s a fixation cross appeared with variable duration (from 1 to 6.5s, mean 2s) until a new trial began.

Two types of stimuli were presented. In rewarded pairs, one of the two stimuli led to reward in 80% of trials, whereas the other stimulus was rewarded on 20% of trials. In bivalent pairs, both stimuli were rewarded on 50% of trials. The same pair of stimuli were presented for 16 successive trials, during which (in the case of the rewarded pairs) the participant had the opportunity to learn, through trial and error, which of the two stimuli was more likely to lead to reward, and earn as much money as possible. After 16 trials, the pair of stimuli changed and the reward contingencies had to be learned from scratch. The task included 10 different pairs of stimuli (i.e. 160 trials), half of them corresponding to rewarded pairs (1st, 3rd, 4th, 5th and 7th) and the other half to bivalent pairs. Subjects were paid the money they won during the experiment. Participants underwent a training session with 48 trials before the scanning session.

We excluded from the analysis participants who chose the optimal stimulus less than 50% of the time during the last eight trials of each rewarded pair (i.e. they had not learnt to choose the rewarded stimulus after 16 trials). Subjects who showed no response or a premature response (reaction time < 100 ms) in more than 10% of the trials were also excluded.

### Computational model

RPE, defined as the difference between the expected and the actual outcome, was estimated using the Q-learning model (Watkins & Dayan, 1992). For each pair of stimuli, the model estimates the expected value of choosing stimulus A (EV_A_) and stimulus B (EV_B_) based on the sequence of choices of subjects. Expected values were set to zero at the beginning of each 16-trial block, and the expected value of the chosen stimulus was updated after each trial as a function of RPE (for further details see online Supplementary Information).

To estimate RPE, the model generates two parameters for each subject, *α* and *β. α* is the rate of learning for the chosen stimulus; it ranges between zero and one and weights the influence of RPE on the updating of expected value. A high *α* value indicates a strong influence of recent outcomes, whereas a low value indicates slow learning. *β* is the (inverse) temperature, a parameter that adjusts the trade-off between exploration and exploitation, i.e. how consistently the model chooses the higher-value stimulus, especially when the values of both stimuli are similar. The higher the *β* value, the more exploratory is the way of choosing; the lower the *β* value, the more deterministic (for further details see online Supplementary Information). The free parameters of the model, *α* and *β,* were estimated for each subject through the maximum likelihood technique to maximize the probability of the actual choices. In the imaging analysis, the learning model was calculated using the same parameters across subjects, through the median of the individual fitted parameters (Daw, 2011).

We carried out two additional analyses, (a) employing RPEs extracted from individually-fitted models instead of using group parameters; and (b) examining brain activation patterns separately for positive and negative RPEs. The latter was with the aim of determining whether negative symptoms were associated with abnormal responses to RPEs of one or both valences (for details of both analyses see online Supplementary Information).

### Image acquisition and pre-processing

Images were acquired with a 3T Philips Ingenia scanner (Philips Medical Systems, Best, The Netherlands). Functional data were acquired using a T2*-weighted echo-planar imaging (EPI) sequence with 443 volumes and the following acquisition parameters: TR = 2000 ms, TE = 30 ms, flip angle = 70°, in-plane resolution = 3.5 × 3.5 mm, FOV = 238 × 245 mm, slice thickness = 3.5 mm, inter-slice gap = 0.75 mm. Slices (32 per volume) were acquired with an interleaved order parallel to the AC-PC plane. In addition, a high-resolution anatomical volume was acquired using an FFE (Fast Field Echo) sequence for anatomical reference and inspection (TR = 9.90 ms; TE = 4.60 ms; Flip angle = 8°; voxel size = 1 × 1 mm; slice thickness = 1 mm; slice number = 180; FOV = 240 mm).

Pre-processing was carried out with the FEAT module included in the FSL (FMRIB Software Library) software (Smith et al., 2004). The first 6 s (3 volumes) of the sequence, corresponding to signal stabilization, were discarded. Pre-processing included motion correction (using the MCFLIRT algorithm), co-registration and normalization to a common stereotactic space (MNI, Montreal Neurological Institute template). For accurate registration, a two-step process was used. First, brain extraction was applied to the structural image, and the functional sequence was registered to it. Then the structural image was registered to the standard template. These two transformations were used to register the functional sequence to the standard space. Before group analyses, normalized images were spatially filtered with a Gaussian filter (FWHM = 5 mm). To minimize unwanted movement-related effects, individuals with an estimated maximum absolute movement >3.0 mm or an average absolute movement >0.3 mm were excluded from the study.

### Statistical analysis

Statistical analysis was performed by means of a General Linear Model (GLM) approach using the FEAT module in FSL. At the first level (within-subject), the following regressors were created: onset of stimuli as 2.5s events in bivalent and reward pairs separately (2 regressors) and onset of feedback as 1s events in bivalent and reward pairs separately (2 regressors). Finally, RPE derived from the computational model was introduced as a parametric modulator at feedback onset as a 1s event across all conditions (one regressor that contained all feedback events across the task, parametrically modulated by the RPE value at each trial). RPE values were signed, meaning that the parametric regressor included both positive and negative RPE values. All regressors were convolved with a gamma response function. We added temporal derivates and motion parameters to the model. Our contrast of interest was the activation associated with the RPE regressor.

GLMs were fitted to generate whole brain individual activation maps for the contrast of interest and second level (group) analyses were performed by means of mixed-effects GLMs (Beckmann, Jenkinson, & Smith, 2003), to obtain mean activation maps for each group. Then, we performed two-sample *t* tests to compare brain activation between the different groups. In all these analyses, whole-brain voxel-wise statistical tests were carried out with a *p* < 0.05, with cluster correction for multiple comparisons, with a cluster-forming threshold of *z* > 3.1 (*p* < 0.001), using Gaussian Random Field correction (Worsley, Marrett, Neelin, & Evans, 1996). In all analyses, age, sex and premorbid IQ were included as covariates in order to minimize the effects of non-significant differences (and in one pairwise comparison a significant difference) among the groups on these variables.

Given the established role of the ventral striatum, orbitofrontal cortex and midbrain in reward processing, we carried out a complementary ROI analysis. Separate right and left ROIs were anatomically defined using the Harvard-Oxford Cortical and Subcortical Atlases (https://fsl.fmrib.ox.ac.uk/fsl/fslwiki/Atlases) for the ventral striatum and orbitofrontal cortex. The midbrain ROI contained the bilateral ventral tegmental area and substantia nigra as defined by Murty et al. (2014).

## Results

From an initial sample of 36 ANS patients, 3 did not complete the task, 3 were excluded due to excessive head motion during the fMRI session, and 3 more due to poor behavioural performance, leaving a final sample of 27 patients. From the initially included 36 HNS patients, 1 did not complete the task and 8 more were excluded, for excessive head motion (3), current IQ < 70 (1), an incidental MRI finding (1), loss of behavioural data due to technical problems (1) and poor behavioural performance (2), leaving 27 patients. Thirty HC were included who were recruited to be similar to the two patient groups for age, sex and TAP-estimated premorbid IQ.

As shown in Table 1, the three groups did not significantly differ in age and sex or TAP-estimated premorbid IQ, although premorbid IQ was significantly lower in the HNS group compared to the HC (*p* = 0.03). As expected, current IQ was significantly lower in both patient groups than in the HC (ANS *v.* HC: *p* = 0.003; HNS *v.* HC: *p* < 0.001), but the two patient groups did not differ significantly on this variable (*p* = 0.56). The patient groups did not significantly differ in the severity of positive (reality distortion) symptoms, illness duration and antipsychotic dose. Both groups were also similar in the type of antipsychotic used: two patients were taking first-generation antipsychotics (one in the ANS and one in the HNS group), 46 were taking second-generation antipsychotics (23 in the ANS and 23 in the HNS group), and 5 were taking both (2 in the ANS group and 3 in the HNS group). Data from pharmacological treatment was missing for one patient in the ANS group. The HNS patients showed higher levels of PANSS disorganization symptoms than the ANS patients, and lower levels of functioning, as measured by the Global Assessment of Functioning scale (GAF).

**Table 1.** Socio-demographic and clinical data in the three groups

	HC (*N* = 30)	ANS (*N* = 27)	HNS (*N* = 27)	Differences
Sex (M/F)	16/14	14/13	20/7	χ^2^ = 3.51 *p* = 0.17
Age	37.33 (13.82)	40.33 (11.57)	41.22 (12.14)	*F* = 0.75 *p* = 0.47
Estimated premorbid IQ	101.90 (8.23)	99.74 (9.37)	96.54 (9.95)	*F* = 2.39 *p* = 0.10
Current IQ	104.83 (12.07)	94.11 (15.10)	91.92 (11.90)	*F* = 7.78 *p* < 0.001
PANSS Total	–	42.00 (8.36)	68.07 (12.90)	*W* = 27 *p* < 0.001
PANSS Positive syndrome	–	8.59 (3.21)	10.59 (4.44)	*W* = 271.5 *p* = 0.107
PANSS Negative syndrome	–	6.78 (0.89)	23.22 (3.26)	Not calculated^a^
PANSS Disorganization	–	4.78 (1.53)	7.19 (3.03)	*W* = 184 *p* = 0.002
CAINS Total	–	10.81 (7.41)	33.15 (7.37)	*W* = 2.5 *p* < 0.001
CAINS MAP	–	10.22 (7.14)	24.37 (5.34)	*W* = 33 *p* < 0.001
CAINS EXP	–	0.59 (1.01)	8.78 (3.45)	*W* = 3 *p* < 0.001
GAF	–	63.59 (17.35)	44.22 (10.20)	*W* = 606 *p* < 0.001
Illness duration (years)	–	15.48 (10.69)	18.33 (11.37)	*W* = 316 *p* = 0.41
Antipsychotic treatment (CPZ equivalents)	–	330.26 (256.06)	409.82 (267.90)	*W* = 270 *p* = 0.15

HC, Healthy controls; ANS, Absent negative symptoms; HNS, High negative symptoms; M/F, Male/Female; PANSS, Positive and Negative Symptoms Scale; CAINS, Clinical Assessment Interview for Negative Symptoms; CAINS-MAP, CAINS motivation and pleasure subscale; CAINS-EXP, CAINS expressivity subscale; GAF, Global Assessment of Functioning; CPZ, chlorpromazine.

Values are means (s.d.). For clinical data, non-parametric tests (Wilcoxon rank sum test) were used, given the non-normal distribution of the data.

a The ANS and HNS patients were a priori selected to differ on PANSS negative symptom scores.

### Behavioural results

**Fig. 2.**
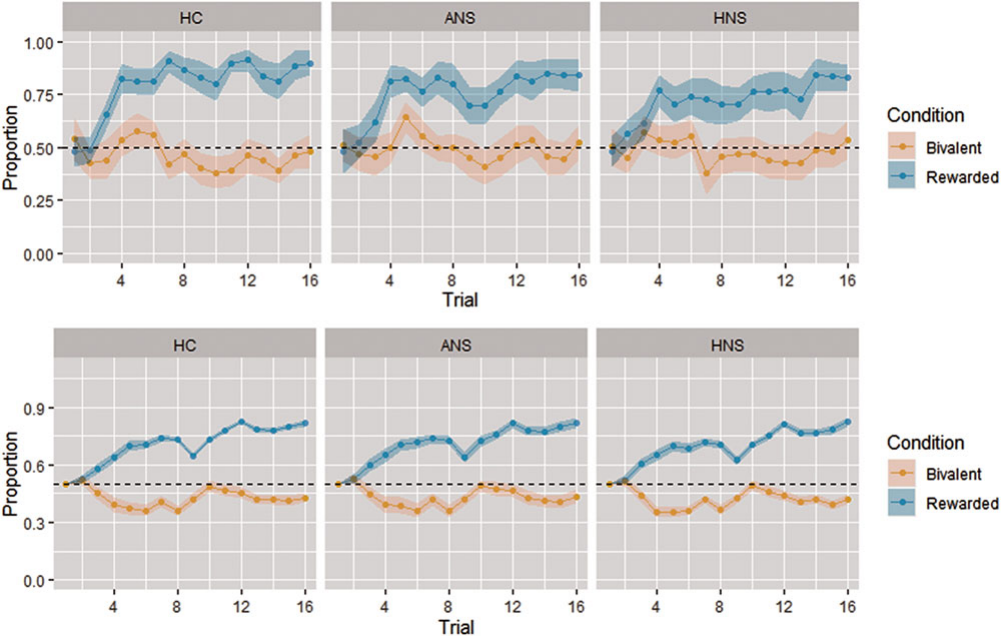
Evolution of responding throughout the task. Upper row plots show the proportion of trials where each group selected the more rewarded stimulus in the rewarded condition (blue line) or one of the two stimuli in the bivalent condition (orange line), in each of the 16 trials that formed each trial block. Lower row plots show the choices predicted by the reinforcement learning model for each group (i.e. the probability of choosing the rewarded stimulus in the rewarded condition, or one of the two stimuli in the bivalent condition). Coloured regions around the lines indicate 95% confidence intervals. The three groups showed evidence of learning in the rewarded condition, since the proportion of choices for the rewarded stimulus progressively increased throughout the trial sequence, although patients (especially in the HNS group) showed a slower increase and did not reach the same proportion of choices than controls. In the bivalent condition, choice proportion remained around 50% throughout the whole trial sequence, as predicted.

The patients showed a lower proportion of correct choices in rewarded pairs (defined as the proportion in which the stimulus with the highest reward probability was chosen, irrespective of the actual outcome) than the HC (HC: 0.80 ± 0.09; ANS: 0.75 ± 0.12; HNS 0.73 ± 0.12, *F* = 3.04, *p* = 0.053), which was significant in the comparison between HC and HNS (*p* = 0.02). For bivalent pairs, there were no group differences in the proportion of choices (HC: 0.46 ± 0.11; ANS: 0.50 ± 0.10; HNS: 0.49 ± 0.08, *F* = 0.83, *p* = 0.44). The three groups showed evidence of learning in the rewarded condition. In the bivalent condition, choice proportions remained around 50% throughout the whole trial sequence for the three groups. The findings are shown in Fig. 2.

The three groups did not differ in the estimates of the Q-learning model parameters *α* (rate of learning) (HC: 0.44 ± 0.33; ANS: 0.43 ± 0.41; HNS: 0.42 ± 0.37, χ^2^ = 0.78, *p* = 0.68) and *β* (temperature) (HC 0.22 ± 0.21; ANS 0.21 ± 0.24; HNS 0.28 ± 0.34, χ^2^ = 1.06, *p* = 0.59). Model fit, measured as the root mean square error (RMSE) of the regression between actual and predicted choices, was similar for HC and ANS patients but was slightly reduced in the HNS patients (*p* < 0.05, see online Supplementary Information for details).

### RPE-associated activations

**Fig. 3.**
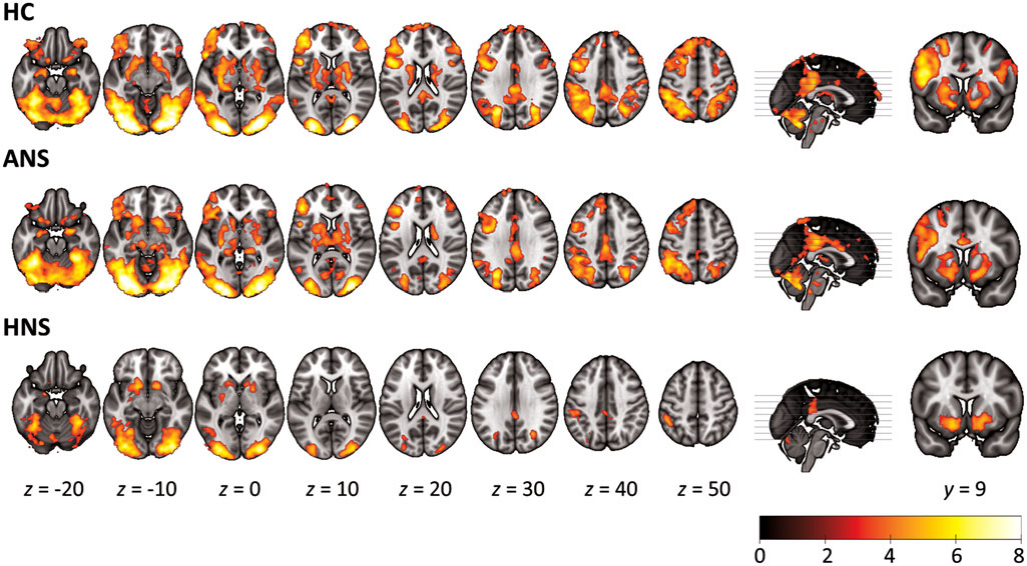
Mean activation maps for positive correlation with RPE in the healthy controls (HC) and the ANS and HNS patients. Coronal slices show ventral striatal activation, present in all three groups. The right side of the image corresponds to the right side of the brain. Colour bar depicts *z* values.

In the HC, RPE value was positively correlated with activation in a wide range of brain regions. These included the ventral striatum and putamen, and portions of the prefrontal cortex, including the bilateral orbitofrontal and ventrolateral prefrontal cortex, the DLPFC (more pronounced on the left) extending into the precentral and medial superior frontal cortex. RPE was also correlated with activation in the posterior cingulate cortex, the bilateral inferior parietal cortex (more pronounced on the left), the bilateral occipital cortex extending into the fusiform gyri, the bilateral amygdala and hippocampus, and the cerebellum (see Fig. 3 and online Supplementary Table S1).

The ANS patients showed a broadly similar pattern of RPE-associated activations, although this appeared less marked in the dorsolateral, inferior frontal, and inferior parietal cortices, particularly on the right. In the HNS patients, regions positively correlated with RPE were limited to the ventral striatum, the bilateral inferior parietal cortex, the posterior cingulate cortex, and the visual cortex extending into the cerebellum.

**Fig. 4.**
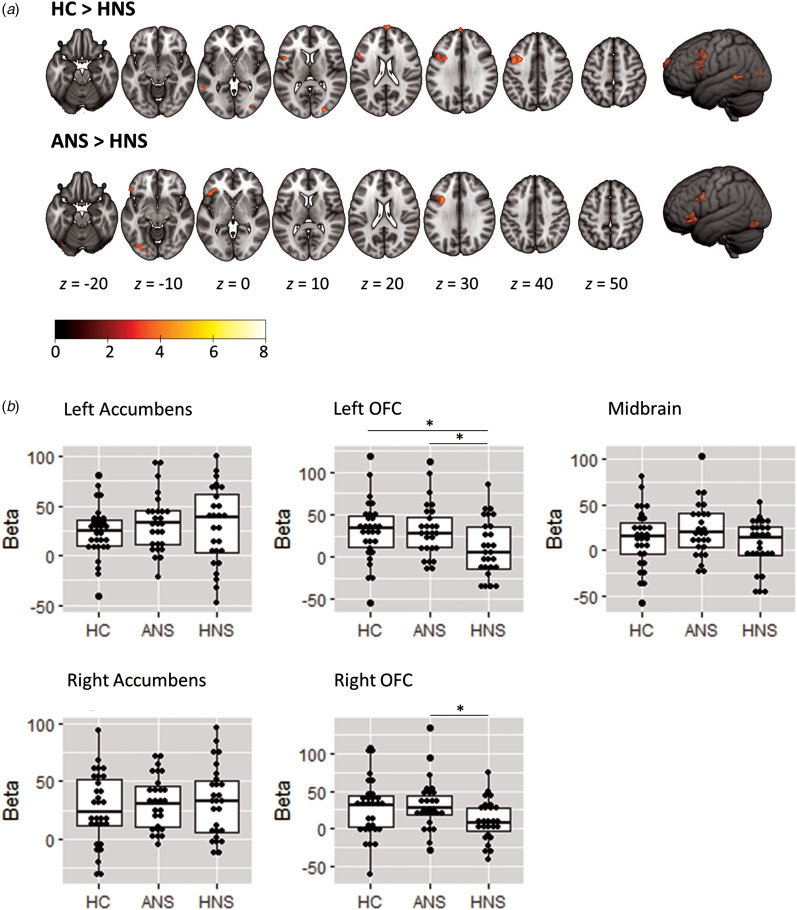
(a) Maps of group comparisons for RPE-associated activations. Top row shows areas of reduced association with RPE for HNS patients relative to heathy controls (HC). Bottom row shows areas of reduced association with RPE for HNS patients relative to ANS patients. The right side of the image corresponds to the right side of the brain. Colour bar depicts *z* values. (b) Scatter plots of RPE-associated activations in ventral striatal, orbitofrontal and midbrain ROIs. Plots show mean parameter estimates (beta values) for the correlation between RPE values and activation within the ROI. The ventral striatum was defined as the nucleus accumbens. The midbrain comprised the ventral tegmental area and substantia nigra bilaterally. OFC, orbitofrontal cortex. * *p* < 0.05.

Group comparisons are shown in Fig. 4a. There were no clusters of significant difference between the HC and the ANS patients. Compared to the HC, the HNS patients showed clusters of significantly reduced RPE-associated activation in the superior medial frontal cortex, the left ventrolateral and dorsolateral prefrontal cortex, the left middle temporal cortex and the bilateral occipital cortex. Compared to the ANS patients, the HNS patients showed reduced activation in the left inferior ventrolateral prefrontal cortex and the DLPFC, and in the left lingual and fusiform gyrus.

Since disorganization scores differed significantly between the HNS and ANS patients, we repeated the whole-brain analysis comparing the two patient groups after covarying for this variable. This caused an additional small cluster of differences to appear in the right inferior parietal cortex (MNI coordinates *x* = 64, *y* = −38, *z* = 22; *Z* value = 4.66; cluster size = 132 voxels; *p* = 0.01).

Examination of RPE-associated activation in the predetermined anatomical ROIs revealed no group differences in the left or right ventral striatum (left: *F* = 0.64, *p* = 0.53; right: *F* = 0.46, *p* = 0.63) (see Fig. 4b). In the left orbitofrontal ROI, there were significant group differences (*F* = 3.28, *p* = 0.04) that reflected a reduced RPE-associated activation in the HNS group relative to the ANS patients (*p* = 0.02) and the HC (*p* = 0.03). The right orbitofrontal ROI showed a similar pattern, but only reaching a trend level (*F* = 2.96, *p* = 0.06). This reflected reduced RPE-related activation in HNS relative to ANS (*p* = 0.02) and HC (*p* = 0.08), with no differences between HC and ANS. The midbrain ROI showed no significant group differences (*F* = 2.00, *p* = 0.14).

### Whole brain findings using other analytic models

Findings employing RPEs extracted from individually-fitted models instead of using group parameters, and examining positive and negative RPEs separately are reported in detail in the online Supplementary Information. Briefly, repeating the whole brain analysis with RPEs extracted from individually-fitted models instead of using group parameters yielded closely similar results to the original analysis. The findings for positive RPEs were also broadly similar to those in the original analysis: the healthy subjects showed clusters of activation in the bilateral ventral striatum and left putamen, the left ventrolateral and dorsolateral prefrontal cortex, the posterior cingulate cortex, the bilateral inferior parietal cortex, the occipital cortex and cerebellum. The pattern was closely similar in the ANS patients, whereas in the HNS patients, activation was seen in the bilateral ventral striatum, the left inferior parietal cortex, the bilateral occipital cortex, and the cerebellum only. Group comparisons showed reduced activation in the HNS patients compared to the ANS patients in the left DLPFC (MNI coordinates: *x* = −42, *y* = 4, *z* = 32; *Z* = 3.94; cluster size = 130 voxels; *p* = 0.01) and left occipital cortex/cerebellum (MNI coordinates: *x* = −38, *y* = −72, *z* = −18; *Z* = 4.53; cluster size = 153 voxels; *p* = 0.006), as shown in online Supplementary Fig. S4. There were no significant group differences in any comparisons for negative RPE associated activations. (For full details and results concerning negative RPEs, see online Supplementary information).

## Discussion

The main finding of this study was that both healthy subjects and schizophrenia patients without negative symptoms showed a broadly similar pattern of activations associated with RPE signalling (albeit slightly less extensive in the latter), whereas activation was reduced in patients with high levels of negative symptoms. Importantly, the areas showing reductions in high negative symptom patients were limited to the dorsolateral and ventrolateral sectors of the prefrontal cortex and did not include the ventral striatum. A further reward-processing- associated region, the orbitofrontal cortex, showed equivocal evidence of reduced activation.

Our findings in healthy subjects show some similarities to those of Garrison, Erdeniz, and Done (2013) in their meta-analysis of studies measuring RPE across a wide variety of functional imaging paradigms. Like us, they found activations affecting large areas of the basal ganglia and other subcortical nuclei such as the thalamus, amygdala and hypothalamus. On the other hand, they found only scattered cortical activations, which took the form principally of a series of discrete clusters in the anterior and middle zones of the medial frontal cortex. More recently, Corlett, Mollick, and Kober (2022) meta-analysed studies of RPE in response to secondary rewards (i.e. money) and found more areas in common with those that we found, including the ventrolateral (and to a small extent the dorsolateral) prefrontal cortex, a large expanse of the medial frontal cortex and the middle/posterior cingulate cortex. Neither meta-analysis found much in the way of occipital cortex activation, but one possible reason for the extensive activations we found in our study might be that our task employed visual stimuli that changed at regular intervals.

Our study failed to find RPE-associated activation differences between patients with high and absent negative symptoms (or between either group or the healthy controls) in the ventral striatum, one of the key target areas for the RPE signal. In this respect, it is noteworthy that three other studies which examined RPE in relation to negative symptoms have not had strong findings in this brain region. One (Katthagen et al., 2020) found that right ventral striatal activation was inversely correlated with negative symptoms; however, the authors appear to have only detected this using a small-volume-corrected voxel-based analysis carried out within a ventral striatal ROI. Another study (Culbreth et al., 2016) found no significant correlation between RPE-related activation and negative symptoms in ventral striatal ROIs in one sample of patients, but a significant inverse correlation on the left in another. The third study (Dowd et al., 2016) found no significant associations between RPE-related activation in striatal ROIs and clinician- or self-rated anhedonia/amotivation.

Our findings for another area importantly involved in reward processing, the orbitofrontal cortex, were equivocal. In the whole brain analysis, there were no differences between patients with high and absent negative symptoms in this region (or between the high negative symptom patients and healthy controls). However, examination of an ROI in the orbitofrontal cortex revealed significantly lower activation in the HNS group on the left and a difference in the same direction on the right. Clearly, a positive finding in an ROI analysis against a background of negative findings in a whole brain analysis needs to be interpreted with caution.

Where we did find evidence of altered RPE-related activation in high negative symptom patients was in the prefrontal cortex, specifically its ventrolateral and dorsolateral regions. Such a finding is of some theoretical interest, as it points to disrupted processing of RPE information in a brain region implicated by the other, ‘impaired frontal function’ theory of negative symptoms. The meaning of such a finding, if replicated in other studies, is uncertain, but one possible interpretation is that altered RPE-related activation in the prefrontal cortex is secondary to a broader prefrontal dysfunctionality underlying negative symptoms, one that would show itself on other tasks as well. To date, functional imaging studies have not consistently found associations between negative symptoms and reduced prefrontal activation during executive (and non-executive) task performance [for a review see Goghari, Sponheim, & MacDonald (2010)]. Recently, however, our group found support for reduced activation in parts of the lateral prefrontal cortex in patients with negative symptoms compared to those without, in a study using a novel paradigm putatively more closely linked to negative symptoms than standard executive tasks (Fuentes-Claramonte et al., 2022).

It is important to point out that our results do not necessarily exclude a role for other aspects of reward processing than RPE in negative symptoms. Probably the main candidate here is reward anticipation, i.e. the motivational properties shown by stimuli that are predictive of reward, a role for dopamine in which has been for argued for persuasively by Berridge (2007). Radua et al. (2015), in their meta-analysis of reward anticipation in the ventral striatum in schizophrenia, found that reduced activation was associated with negative symptoms, although the number of studies was small (*N* = 6) and the association was only seen on the left. Subsequent studies have had inconsistent findings: apathy/amotivation was found to be inversely correlated with ventral striatal activation during reward anticipation in three studies (Kirschner et al., 2016; Kluge et al., 2018; Simon et al., 2015), but this was only equivocally the case in a fourth (Wolf et al., 2014). Another study (Moran, Culbreth, Kandala, & Barch, 2019) found a significant inverse correlation between the ‘motivation and pleasure’ subscale of the CAINS and activation in the anterior cingulate cortex and insula.

In conclusion, our study does not suggest that a general dysfunction in RPE signalling underlies negative symptoms, but does find evidence for one that is restricted to the lateral prefrontal cortex. Some limitations of our study need to be acknowledged. At 54, the numbers of patients included was larger than some other studies of RPE in relation to negative symptoms, but the high and absent negative symptom groups were still individually relatively small. All the patients in the study were taking antipsychotic medication: while this may have influenced the findings in patients compared to healthy controls, it seems unlikely that this factor would have played a role in the differences found between patients with and without negative symptoms. Finally, the results in the online Supplementary analysis of positive and negative RPEs found evidence of an abnormality in relation to positive RPEs, but were weaker than in the main analysis.

## Supporting information

Fuentes-Claramonte et al. supplementary material Fuentes-Claramonte et al. supplementary material

## Supplementary material

The supplementary material for this article can be found at https://doi.org/10.1017/S0033291723000521

## References

[ref1] Beckmann, C. F., Jenkinson, M., & Smith, S. M. (2003). General multilevel linear modeling for group analysis in FMRI. NeuroImage, 20, 1052–1063. 14568475 10.1016/S1053-8119(03)00435-X

[ref2] Berridge, K. C. (2007). The debate over dopamine's role in reward: The case for incentive salience. Psychopharmacology, 191, 391–431. 17072591 10.1007/s00213-006-0578-x

[ref3] Bucci, P., & Galderisi, S. (2017). Categorizing and assessing negative symptoms. Current Opinion in Psychiatry, 30, 201–208. 28212174 10.1097/YCO.0000000000000322

[ref200] Chase H. W., Loriemi P., Wensing T., Eickhoff S. B., & Nickl-Jockschat T. (2018). Meta-analytic evidence for altered mesolimbic responses to reward in schizophrenia. Human Brain Mapping, 39, 2917–2928. 29573046 10.1002/hbm.24049 PMC6866586

[ref4] Corlett, P. R., Mollick, J. A., & Kober, H. (2022). Meta-analysis of human prediction error for incentives, perception, cognition, and action. Neuropsychopharmacology, 47, 1339–1349. 35017672 10.1038/s41386-021-01264-3 PMC9117315

[ref5] Culbreth, A. J., Westbrook, A., Xu, Z., Barch, D. M., & Waltz, J. A. (2016). Intact ventral striatal prediction error signaling in medicated schizophrenia patients. Biological Psychiatry: Cognitive Neuroscience and Neuroimaging, 1, 474–483. 28239676 10.1016/j.bpsc.2016.07.007 PMC5321567

[ref6] Davis, K. L., Kahn, R. S., Ko, G., & Davidson, M. (1991). Dopamine in schizophrenia: A review and reconceptualization. American Journal of Psychiatry, 148, 1474–1486. 1681750 10.1176/ajp.148.11.1474

[ref7] Daw, N. D. (2011). Trial-by-trial data analysis using computational models. In M. R. Delgado, E. A. Phelps & T. W. Robbins (Eds.), Decision making, affect, and learning: Attention and performance XXIII (pp. 3–38). Oxford: Oxford University Press.

[ref8] de Gracia Dominguez, M., Viechtbauer, W., Simons, C. J., van Os, J., & Krabbendam, L. (2009). Are psychotic psychopathology and neurocognition orthogonal? A systematic review of their associations. Psychological Bulletin, 135, 157–171. 19210058 10.1037/a0014415

[ref9] Del Ser, T., Gonzalez-Montalvo, J. I., Martinez-Espinosa, S., Delgado-Villapalos, C., & Bermejo, F. (1997). Estimation of premorbid intelligence in Spanish people with the Word Accentuation Test and its application to the diagnosis of dementia. Brain and Cognition, 33, 343–356. 9126399 10.1006/brcg.1997.0877

[ref10] Deserno, L., Boehme, R., Heinz, A., & Schlagenhauf, F. (2013). Reinforcement learning and dopamine in schizophrenia: Dimensions of symptoms or specific features of a disease group? Frontiers in Psychiatry, 4, 172. 24391603 10.3389/fpsyt.2013.00172 PMC3870301

[ref11] Dibben, C. R., Rice, C., Laws, K., & McKenna, P. J. (2009). Is executive impairment associated with schizophrenic syndromes? A meta-analysis. Psychological Medicine, 39, 381–392. 18588741 10.1017/S0033291708003887

[ref12] Diekhof, E. K., Kaps, L., Falkai, P., & Gruber, O. (2012). The role of the human ventral striatum and the medial orbitofrontal cortex in the representation of reward magnitude – an activation likelihood estimation meta-analysis of neuroimaging studies of passive reward expectancy and outcome processing. Neuropsychologia, 50, 1252–1266. 22366111 10.1016/j.neuropsychologia.2012.02.007

[ref13] Dowd, E. C., Frank, M. J., Collins, A., Gold, J. M., & Barch, D. M. (2016). Probabilistic reinforcement learning in patients with schizophrenia: Relationships to anhedonia and avolition. Biological Psychiatry: Cognitive Neuroscience and Neuroimaging, 1, 460–473. 27833939 10.1016/j.bpsc.2016.05.005 PMC5098503

[ref14] Fervaha, G., Foussias, G., Agid, O., & Remington, G. (2014). Impact of primary negative symptoms on functional outcomes in schizophrenia. European Psychiatry, 29, 449–455. 24630742 10.1016/j.eurpsy.2014.01.007

[ref15] First, M. B., Spitzer, R. L., Gibbon, M., & Williams, J. B. W. (2002). The structured clinical interview for DSM-IV axis I disorders *(*research version*)*. New York: Biometrics Research, New York State Psychiatric Institute.

[ref16] Frith, C. D. (1992). The cognitive neuropsychology of schizophrenia. Hove: Erlbaum (UK) Taylor & Francis.

[ref17] Fuentes-Claramonte, P., Ramiro, N., Torres, L., Argila-Plaza, I., Salgado-Pineda, P., Soler-Vidal, J., … Pomarol-Clotet, E. (2022). Negative schizophrenic symptoms as prefrontal cortex dysfunction: Examination using a task measuring goal neglect. NeuroImage Clinical, 35, 103119. 35870381 10.1016/j.nicl.2022.103119 PMC9421442

[ref18] Garrison, J., Erdeniz, B., & Done, J. (2013). Prediction error in reinforcement learning: A meta-analysis of neuroimaging studies. Neuroscience and Biobehavioral Reviews, 37, 1297–1310. 23567522 10.1016/j.neubiorev.2013.03.023

[ref19] Goghari, V. M., Sponheim, S. R., & MacDonald, A. W. III. (2010). The functional neuroanatomy of symptom dimensions in schizophrenia: A qualitative and quantitative review of a persistent question. Neuroscience and Biobehavioral Reviews, 34, 468–486. 19772872 10.1016/j.neubiorev.2009.09.004 PMC2813961

[ref20] Gomar, J. J., Ortiz-Gil, J., McKenna, P. J., Salvador, R., Sans-Sansa, B., Sarro, S., … Pomarol-Clotet, E. (2011). Validation of the Word Accentuation Test (TAP) as a means of estimating premorbid IQ in Spanish speakers. Schizophrenia Research, 128, 175–176. 21144711 10.1016/j.schres.2010.11.016

[ref21] Howes, O. D., & Kapur, S. (2009). The dopamine hypothesis of schizophrenia: Version III – the final common pathway. Schizophrenia Bulletin, 35, 549–562. 19325164 10.1093/schbul/sbp006 PMC2669582

[ref22] Jauhar, S., Fortea, L., Solanes, A., Albajes-Eizagirre, A., McKenna, P. J., & Radua, J. (2021). Brain activations associated with anticipation and delivery of monetary reward: A systematic review and meta-analysis of fMRI studies. PLoS One, 16, e0255292. 34351957 10.1371/journal.pone.0255292 PMC8341642

[ref23] Katthagen, T., Kaminski, J., Heinz, A., Buchert, R., & Schlagenhauf, F. (2020). Striatal dopamine and reward prediction error signaling in unmedicated schizophrenia patients. Schizophrenia Bulletin, 46, 1535–1546. 32318717 10.1093/schbul/sbaa055 PMC7751190

[ref24] Kirkpatrick, B. (2014). Progress in the study of negative symptoms. Schizophrenia Bulletin, 40(Suppl 2), S101–S106. 24562490 10.1093/schbul/sbt158 PMC3934401

[ref25] Kirschner, M., Hager, O. M., Bischof, M., Hartmann, M. N., Kluge, A., Seifritz, E., … Kaiser, S. (2016). Ventral striatal hypoactivation is associated with apathy but not diminished expression in patients with schizophrenia. Journal of Psychiatry & Neuroscience: JPN, 41, 152–161. 26395814 10.1503/jpn.140383 PMC4853206

[ref26] Kluge, A., Kirschner, M., Hager, O. M., Bischof, M., Habermeyer, B., Seifritz, E., … Kaiser, S. (2018). Combining actigraphy, ecological momentary assessment and neuroimaging to study apathy in patients with schizophrenia. Schizophrenia Research, 195, 176–182. 29030262 10.1016/j.schres.2017.09.034

[ref27] Kring, A. M., Gur, R. E., Blanchard, J. J., Horan, W. P., & Reise, S. P. (2013). The Clinical Assessment Interview for Negative Symptoms (CAINS): Final development and validation. The American Journal of Psychiatry, 170, 165–172. 23377637 10.1176/appi.ajp.2012.12010109 PMC3785242

[ref201] Leroy A., Amad A., D'Hondt F., Pins D., Jaafari N., Thomas P., & Jardri P. (2020). Reward anticipation in schizophrenia: A coordinate-based meta-analysis. Schizophrenia Research, 218, 2–6. 31948895 10.1016/j.schres.2019.12.041

[ref28] Liddle, P. F. (1987). Schizophrenic syndromes, cognitive performance and neurological dysfunction. Psychological Medicine, 17, 49–57. 3575577 10.1017/s0033291700012976

[ref29] Liu, X., Hairston, J., Schrier, M., & Fan, J. (2011). Common and distinct networks underlying reward valence and processing stages: A meta-analysis of functional neuroimaging studies. Neuroscience and Biobehavioral Reviews, 35, 1219–1236. 21185861 10.1016/j.neubiorev.2010.12.012 PMC3395003

[ref30] Moran, E. K., Culbreth, A. J., Kandala, S., & Barch, D. M. (2019). From neuroimaging to daily functioning: A multimethod analysis of reward anticipation in people with schizophrenia. Journal of Abnormal Psychology, 128, 723–734. 31464449 10.1037/abn0000461 PMC6776676

[ref31] Murty, V. P., Shermohammed, M., Smith, D. V., Carter, R. M., Huettel, S. A., & Adcock, R. A. (2014). Resting state networks distinguish human ventral tegmental area from substantia nigra. NeuroImage, 100, 580–589. 24979343 10.1016/j.neuroimage.2014.06.047 PMC4370842

[ref32] Oldham, S., Murawski, C., Fornito, A., Youssef, G., Yucel, M., & Lorenzetti, V. (2018). The anticipation and outcome phases of reward and loss processing: A neuroimaging meta-analysis of the monetary incentive delay task. Human Brain Mapping, 39, 3398–3418. 29696725 10.1002/hbm.24184 PMC6055646

[ref33] Pessiglione, M., Seymour, B., Flandin, G., Dolan, R. J., & Frith, C. D. (2006). Dopamine-dependent prediction errors underpin reward-seeking behaviour in humans. Nature, 442, 1042–1045. 16929307 10.1038/nature05051 PMC2636869

[ref34] Radua, J., Schmidt, A., Borgwardt, S., Heinz, A., Schlagenhauf, F., McGuire, P., & Fusar-Poli, P. (2015). Ventral striatal activation during reward processing in psychosis: A neurofunctional meta-analysis. JAMA Psychiatry, 72, 1243–1251. 26558708 10.1001/jamapsychiatry.2015.2196

[ref35] Schultz, W., Dayan, P., & Montague, P. R. (1997). A neural substrate of prediction and reward. Science (New York, N.Y.), 275, 1593–1599. 9054347 10.1126/science.275.5306.1593

[ref36] Simon, J. J., Cordeiro, S. A., Weber, M. A., Friederich, H. C., Wolf, R. C., Weisbrod, M., & Kaiser, S. (2015). Reward system dysfunction as a neural substrate of symptom expression across the general population and patients with schizophrenia. Schizophrenia Bulletin, 41, 1370–1378. 26006262 10.1093/schbul/sbv067 PMC4601714

[ref37] Smith, S. M., Jenkinson, M., Woolrich, M. W., Beckmann, C. F., Behrens, T. E., Johansen-Berg, H., … Matthews, P. M. (2004). Advances in functional and structural MR image analysis and implementation as FSL. NeuroImage, 23(Suppl 1), S208–S219. 15501092 10.1016/j.neuroimage.2004.07.051

[ref38] Valiente-Gomez, A., Mezquida, G., Romaguera, A., Vilardebo, I., Andres, H., Granados, B., … Bernardo, M. (2015). Validation of the Spanish version of the Clinical Assessment for Negative Symptoms (CAINS). Schizophrenia Research, 166, 104–109. 26116328 10.1016/j.schres.2015.06.006

[ref39] Wallwork, R. S., Fortgang, R., Hashimoto, R., Weinberger, D. R., & Dickinson, D. (2012). Searching for a consensus five-factor model of the positive and negative syndrome scale for schizophrenia. Schizophrenia Research, 137, 246–250. 22356801 10.1016/j.schres.2012.01.031 PMC3351536

[ref40] Watkins, C. J., & Dayan, P. (1992). Q-learning. Machine Learning, 8, 279–292.

[ref41] Weinberger, D. R. (1988). Schizophrenia and the frontal lobe. Trends in Neurosciences, 11, 367–370. 2469198 10.1016/0166-2236(88)90060-4

[ref42] Wolf, D. H., Satterthwaite, T. D., Kantrowitz, J. J., Katchmar, N., Vandekar, L., Elliott, M. A., & Ruparel, K. (2014). Amotivation in schizophrenia: Integrated assessment with behavioral, clinical, and imaging measures. Schizophrenia Bulletin, 40, 1328–1337. 24657876 10.1093/schbul/sbu026 PMC4193711

[ref43] Worsley, K. J., Marrett, S., Neelin, P., & Evans, A. C. (1996). A unified statistical approach for determining significant signals in location and scale space images of cerebral activation. In R. Myers, V. Cunningham, D. Bailey & T. Jones (Eds.), Quantification of brain function using PET (pp. 327–333). Oxford: Academic Press.

[ref44] Yaple, Z. A., Tolomeo, S., & Yu, R. (2021). Abnormal prediction error processing in schizophrenia and depression. Human Brain Mapping, 42, 3547–3560. 33955106 10.1002/hbm.25453 PMC8249895

[ref45] Yarkoni, T. (2009). Big correlations in little studies: Inflated fMRI correlations reflect low statistical power-commentary on Vul *et al*. (2009). Perspectives on Psychological Science, 4, 294–298. 26158966 10.1111/j.1745-6924.2009.01127.x

[ref46] Zeng, J., Yan, J., Cao, H., Su, Y., Song, Y., Luo, Y., & Yang, X. (2022). Neural substrates of reward anticipation and outcome in schizophrenia: A meta-analysis of fMRI findings in the monetary incentive delay task. Translational Psychiatry, 12, 448. 36244990 10.1038/s41398-022-02201-8 PMC9573872

