# Supplementary material for: Do the negative symptoms of schizophrenia reflect reduced responsiveness to reward? Examination using a reward prediction error (RPE) task
Source: Psychol Med. 2023 Mar 29;53(15):7106–15. doi: 10.1017/S0033291723000521 (PMC10719670; doi:10.1017/S0033291723000521)
Supplement: Fuentes-Claramonte et al. supplementary material [file S0033291723000521sup001.docx]

**Supplementary information for: Do the negative symptoms of schizophrenia reflect altered responsiveness to reward? Examination using a reward prediction error (RPE) task. Fuentes-Claramonte et al.**

**Supplementary Methods**

*Computational model for main analysis*

RPE was estimated using the Q-learning model (Watkins and Dayan, 1992). In this, for each pair of stimuli, the model estimates the expected value of choosing stimulus A (EV_A_) and stimulus B (EV_B_) based on the sequence of choices of subjects. Expected values were set to zero at the beginning of each 16-trial block, and the expected value of the chosen stimulus was updated after each trial as a function of RPE. As an example, if the subject chooses the stimulus *A*:

$${RPE}_{t}=R_{t}-{EV}_{A,t}$$

$${EV}_{A,t+1}={EV}_{A,t}+\alpha*{RPE}_{t}$$

where *RPE_t_* is the reward prediction error at time *t* (i.e., the trial that has just finished), *R_t_* is the reward at time *t*, *EV_A,t_* is the expected value of *A* (the chosen stimulus) at time *t*, *EV_A,t_*_+1_ is the expected value of *A* at time *t*+1 (i.e., the following trial), and the parameter *α* is the rate of learning for the chosen stimulus. This parameter, which ranges between zero and one, weights the influence of RPE on the updating of expected value. A high *α* value indicates a strong influence of recent outcomes, whereas a low *α* value indicates a slow learning.

The model also estimates the trial-by-trial probability of choosing each stimulus based on the expected values using a softmax function (Gläscher and O'Doherty, 2010). As an example, the probability of choosing stimulus A:

$$p_{A}=\frac{{{EV}_{A}}/\beta}{\left( {{EV}_{A}}/\beta\right)+\left( {{EV}_{B}}/\beta\right)}$$

where *β* is the (inverse) temperature, a parameter that adjusts the trade-off between exploration and exploitation, i.e. how consistently the model chooses the higher-value stimulus, especially when the values of both stimuli are similar. The higher the *β* value, the more exploratory is the way of choosing; the lower the *β* value, the more deterministic.

We fitted the model based on the sequence of choices of subjects. The free parameters of the model, *α* and *β,* were estimated for each subject through the maximum likelihood technique to maximize the probability of the actual choices. For the behavioural analysis, individual parameter estimates were compared between groups using the non-parametric Kruskal-Wallis test, due to the non-normal distribution of the data. For the imaging analysis, the learning model was calculated using the same parameters across subjects, through the median of the individual fitted parameters (Daw, 2011). We conducted all behavioural and computational analyses in R 3.6.1 (R Core Team, 2022).

**Supplementary results**

*Computational model fit*

We tested the fit of the computational model by regressing, for each subject, the actual trial-by-trial choices throughout the task (which stimulus was selected in each trial) vs the choice predicted by the model (the probability that the model estimated for selecting the same stimulus). We used the root mean square error (RMSE) of the regression model as a measure of model fit for each participant. RMSE values were highly similar for HC (mean RMSE = 0.37±0.08) and ANS patients (mean RMSE = 0.37±0.06), but slightly higher for HNS patients (mean RMSE = 0.42±0.07). Pairwise comparisons (t-tests) showed that model fit for HNS patients differed from HC (*p* = 0.02) and from ANS patients (*p* = 0.02), while ANS patients and HC had equivalent values (*p* = 0.99).

*RPE association with brain activity*

Our main analysis examined the association between brain activity and RPE, calculated through a Q-learning model that used fixed values for the *α* and *β* parameters (median of the total sample). Complementary to this approach, we ran an additional analysis with RPEs extracted from the model with individually fitted parameters for each subject. The brain regions associated with RPE were found to be broadly to those identified using the group parameters (see Supplementary Figure 1): for HC, RPE values were positively correlated with activity in the ventral striatum and putamen, the bilateral orbitofrontal and ventrolateral prefrontal cortex, the DLPFC (especially the left), the precentral gyrus and the medial prefrontal cortex, and also the posterior cingulate, inferior parietal cortex (bilateral but more pronounced on the left), occipital cortex, fusiform gyri, amygdala, hippocampus and cerebellum. The activation map was similar for ANS patients, but with less marked activation in fronto-parietal regions, while in the HNS patients activation linked to RPE was limited to the ventral striatum, inferior parietal cortex, posterior cingulate, visual cortex and cerebellum, with a small activation cluster in the right inferior frontal cortex.


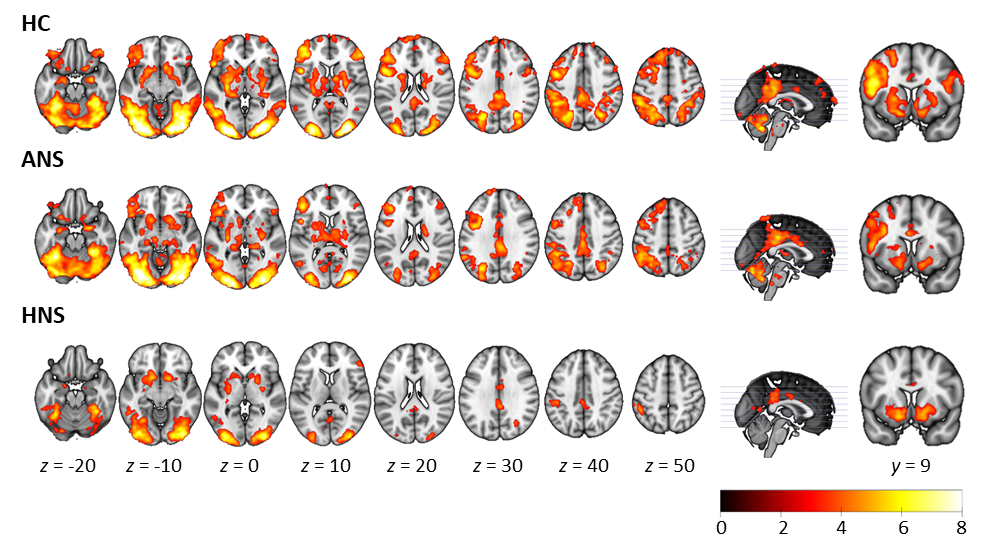


**Supplementary Figure 1.** Mean activation maps for positive correlation with RPEs extracted from the individually fitted model in the healthy controls (HC) and the ANS and HNS patients. Coronal slices show ventral striatal activation, present in all three groups. The right side of the image corresponds to the right side of the brain. Colour bar depicts *z* values.

Group comparisons (shown in Supplementary Figure 2) showed no differences between the HC and the ANS patients. Relative to the HC, the HNS patients showed reduced activation in the left DLPFC (MNI coordinates: *x* = -32, *y* = 8, *z* = 38; Z = 4.36; cluster size = 288 voxels; *p* < 0.001) and the right occipital cortex (MNI coordinates: *x* = 28, *y* = -90, *z* = 10; Z = 4.49; cluster size = 143 voxels; *p* = 0.007). Compared to the ANS patients, the HNS patients showed reduced activation in the left inferior ventrolateral prefrontal cortex (MNI coordinates: *x* = -50, *y* = 22, *z* = -4; Z = 4.27; cluster size = 166 voxels; *p* = 0.003), the left DLPFC (MNI coordinates: *x* = -32, *y* = 12, *z* = 30; Z = 4.30; cluster size = 149 voxels; *p* = 0.005) and the left occipital cortex (MNI coordinates: *x* = -32, *y* = -80, *z* = -12; Z = 4.38; cluster size = 137 voxels; *p* = 0.008).


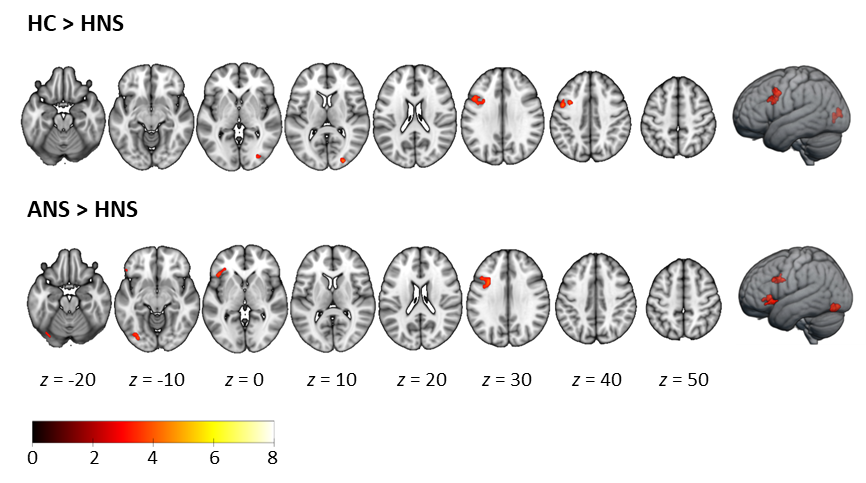


**Supplementary Figure 2.** Maps for group comparisons for RPE-associated activations, extracted from the individually fitted model. Top row shows areas of reduced association with RPE for HNS patients relative to controls. Bottom row shows areas of reduced association with RPE for HNS patients relative to ANS patients. The right side of the image corresponds to the right side of the brain. Colour bar depicts *z* values.

*Brain activity associated with positive and negative RPE*

In this additional complementary analysis, we examined brain activations associated with positive and negative RPEs separately, with the aim of determining whether negative symptoms showed abnormal responses to RPEs of one or both valences. Mean activation maps for positive and negative RPEs in each group are shown in the Supplementary Figure 3. In the HC, positive RPEs were associated with activation in the bilateral ventral striatum and left putamen, the left ventrolateral and dorsolateral prefrontal cortex, the posterior cingulate cortex, and the bilateral inferior parietal cortex, occipital cortex and cerebellum. Negative RPEs were associated with activation in the bilateral orbitofrontal and ventrolateral PFC, the frontal pole, the left putamen, the posterior cingulate and bilateral occipital cortex and the cerebellum. The ANS patients showed a very similar activation pattern, although putamen activation was bilateral for positive RPEs, and for negative RPEs activation was more restricted and spanned the left orbitofrontal and bilateral ventrolateral cortex, the medial prefrontal cortex/ACC and the frontal pole, the left inferior parietal cortex and the bilateral occipital cortex and cerebellum. In the HNS patients, activation was seen in the bilateral ventral striatum, the left inferior parietal, the bilateral occipital cortex and the cerebellum for positive RPEs. For negative RPEs, activation was limited to the visual cortex and cerebellum.

Group comparisons revealed differences between the ANS and the HNS patients. As shown in Supplementary Figure 4., the HNS patients showed reduced activation associated with positive RPE in the left DLPFC (MNI coordinates: *x* = -42, *y* = 4, *z* = 32; Z = 3.94; cluster size = 130 voxels; *p* = 0.013) and the left occipital cortex/cerebellum (MNI coordinates: *x* = -38, *y* = -72, *z* = -18; Z = 4.53; cluster size = 153 voxels; *p* = 0.006), There were no other group differences between the HC and the two patient groups for either positive or negative RPEs.


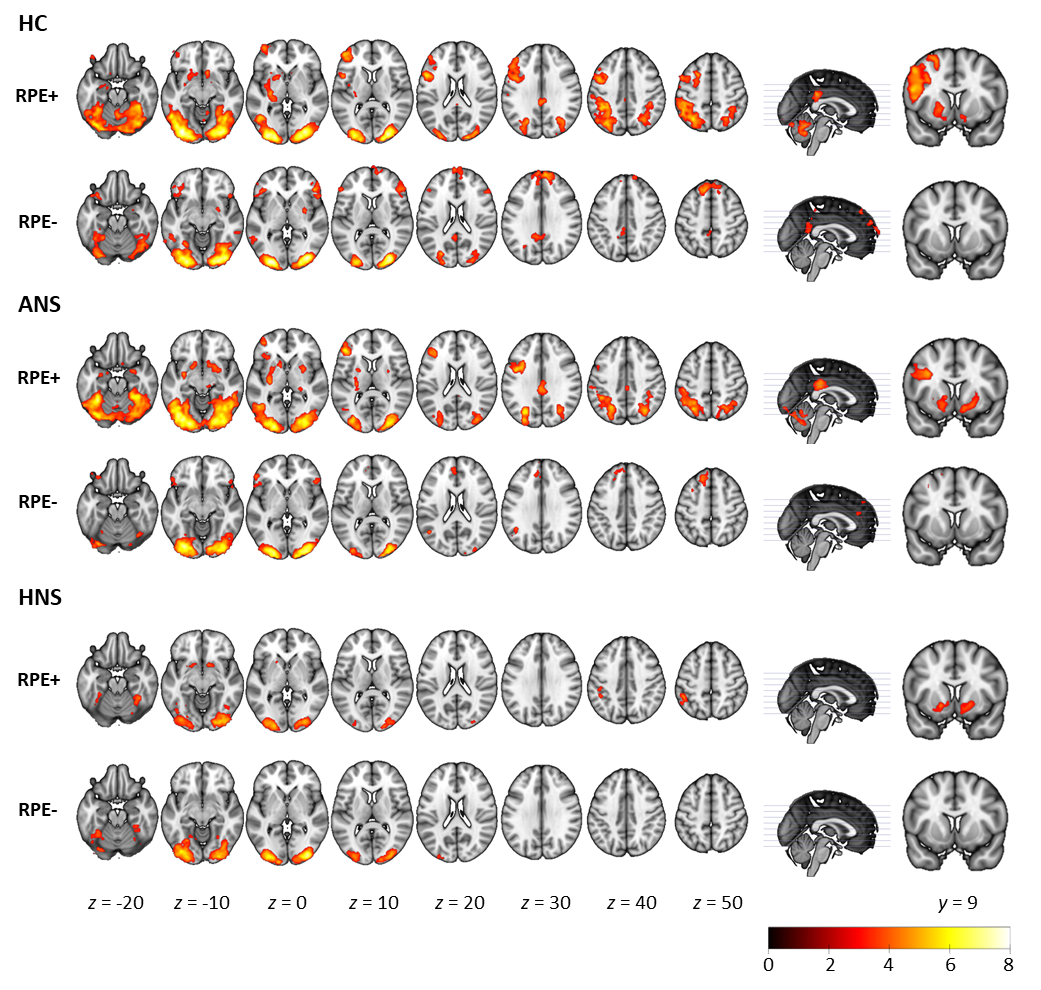


**Supplementary Figure 3.** Mean activation maps for positive correlation with positive RPEs (RPE+) and negative RPEs (RPE-) in the healthy controls (HC) and the ANS and HNS patients. Coronal slices show ventral striatal activation, present in the three groups for positive RPEs. The right side of the image corresponds to the right side of the brain. Colour bar depicts *z* values.


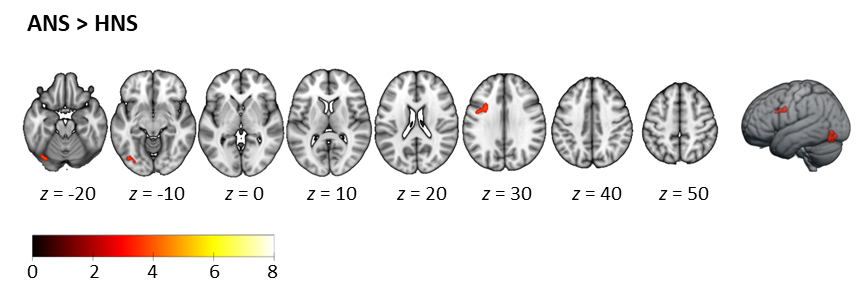


**Supplementary Figure 4.** Map for significant group differences for activation associated to positive RPEs, showing regions of reduced activation in the HNS group relative to the ANS group. The right side of the image corresponds to the right side of the brain. Colour bar depicts *z* values.

**Supplementary references**

**Daw ND** (2011). Trial-by-trial data analysis using computational models. In *Decision Making, Affect, and Learning: Attention and Performance xxiii* (ed. M. R. Delgado, E. A. Phelps and T. W. Robbins), pp. 3-38. Oxford University Press: Oxford.

**Gläscher JP & O'Doherty JP (2010).** Model-based approaches to neuroimaging: Combining reinforcement learning theory with fMRI data. *Wiley Interdisciplinary Reviews Cognitive Science* **1,** 501-510.

**R Core Team** (2022). R: A language and environment for statistical computing. R Foundation for Statistical Computing: Vienna.

**Watkins CJ & Dayan P** (1992). Q-learning. *Machine Learning* **8** 279-292.

**Supplementary table 1.** Peaks of activation associated with RPE in each group.

|  | **MNI coordinates** | | |  |  |  |
| --- | --- | --- | --- | --- | --- | --- |
| **Region** | ***x*** | ***y*** | ***z*** | ***Z*** | ***k*** | ***p*** |
| *Controls (HC)* |  |  |  |  |  |  |
| Middle occipital cortex | 28 | -90 | 8 | 9.80 | 32498 | <0.001 |
|  | -30 | -92 | 0 | 9.35 |  |  |
| Inferior occipital cortex | -34 | -84 | -8 | 9.48 |  |  |
|  | 34 | -88 | 2 | 9.30 |  |  |
| Lingual gyrus | -26 | -88 | -14 | 9.19 |  |  |
| Fusiform gyrus | 36 | -72 | -14 | 8.04 |  |  |
|  | -38 | -72 | -16 | 8.01 |  |  |
| Calcarine cortex | 18 | -96 | 4 | 7.99 |  |  |
| Inferior temporal cortex | -52 | -58 | -10 | 7.42 |  |  |
| Precentral gyrus | -48 | 6 | 36 | 6.90 | 10941 | <0.001 |
| IFG, *pars opercularis* | -44 | 8 | 24 | 6.87 |  |  |
| IFG, *pars triangularis* | -40 | 40 | 12 | 6.84 |  |  |
| DLPFC | -42 | 48 | 10 | 6.49 |  |  |
| IFG, *pars orbitalis* | -52 | 22 | -2 | 6.39 |  |  |
| Superior frontal gyrus | -22 | 14 | 54 | 6.12 |  |  |
| OFC | -44 | 54 | -6 | 5.90 |  |  |
| Medial superior frontal cortex | -10 | 38 | 48 | 5.54 |  |  |
| Ventral striatum | -14 | 10 | -14 | 6.05 | 6696 | <0.001 |
|  | 12 | 14 | -14 | 5.77 |  |  |
| Pallidum | -20 | 6 | 4 | 5.63 |  |  |
| Hippocampus | 28 | -2 | -22 | 5.59 |  |  |
|  | -30 | -6 | -14 | 4.82 |  |  |
| Putamen | 30 | -8 | 6 | 5.58 |  |  |
|  | -26 | -6 | 4 | 5.42 |  |  |
| Parahippocampal cortex | -18 | -4 | -24 | 5.27 |  |  |
| Amygdala | 26 | -2 | -14 | 5.06 |  |  |
| Thalamus | 18 | -30 | 10 | 5.05 |  |  |
| IFG, *pars orbitalis* | 20 | 16 | -24 | 4.74 |  |  |
| Caudate | 8 | 16 | -6 | 4.67 |  |  |
| IFG, *pars triangularis* | 54 | 38 | 14 | 6.13 | 2436 | <0.001 |
| IFG, *pars orbitalis* | 40 | 42 | -16 | 5.54 |  |  |
| DLPFC | 40 | 28 | 42 | 4.87 |  |  |
| IFG, *pars opercularis* | 48 | 8 | 24 | 4.75 |  |  |
| Precentral gyrus | 64 | 8 | 26 | 4.13 |  |  |
| Posterior cingulate cortex | -4 | -32 | 32 | 5.64 | 2226 | <0.001 |
| Middle cingulate cortex | -4 | -36 | 44 | 5.20 |  |  |
| Precuneus | 0 | -48 | 36 | 4.94 |  |  |
| Calcarine cortex | 2 | -60 | 14 | 3.60 |  |  |
| Superior frontal gyrus | 30 | 68 | 4 | 4.78 | 256 | <0.001 |
| Precentral gyrus | 26 | -6 | 48 | 4.07 | 243 | <0.001 |
|  | 30 | -24 | 56 | 4.25 | 113 | 0.026 |
| ACC | -4 | 6 | 28 | 4.25 | 144 | 0.008 |
|  |  |  |  |  |  |  |
| *ANS patients* |  |  |  |  |  |  |
| Inferior occipital cortex | -34 | -84 | -10 | 9.19 | 44087 | <0.001 |
|  | 36 | -84 | -10 | 8.63 |  |  |
| Middle occipital cortex | 28 | -90 | 8 | 8.97 |  |  |
|  | -32 | -90 | -4 | 8.39 |  |  |
| Fusiform gyrus | -36 | -74 | -12 | 8.84 |  |  |
|  | 34 | -66 | -14 | 7.63 |  |  |
| Lingual gyrus | 22 | -88 | -8 | 8.72 |  |  |
|  | -26 | -88 | -12 | 8.71 |  |  |
| Cerebellum | -26 | -52 | -16 | 7.10 |  |  |
| IFG, *pars triangularis* | -46 | 36 | 10 | 6.70 |  |  |
| IFG, *pars orbitalis* | -50 | 22 | -4 | 6.28 |  |  |
| Inferior parietal cortex | -46 | -54 | 48 | 6.24 |  |  |
| Superior parietal cortex | -28 | -54 | 50 | 6.16 |  |  |
| Middle temporal cortex | -50 | -60 | -2 | 6.08 |  |  |
| Precentral gyrus | -42 | 2 | 28 | 6.00 |  |  |
| Middle cingulate gyrus | 0 | -28 | 38 | 5.97 |  |  |
| IFG, *pars orbitalis* | 50 | 22 | -8 | 5.10 | 505 | <0.001 |
| DLPFC | 40 | 50 | 22 | 4.14 | 341 | <0.001 |
| ACC | -4 | 48 | 14 | 4.15 | 170 | 0.003 |
| Superior frontal gyrus | -16 | 58 | 28 | 4.33 | 166 | 0.004 |
|  |  |  |  |  |  |  |
| *HNS patients* |  |  |  |  |  |  |
| Inferior occipital cortex | -36 | -84 | -10 | 7.39 | 4779 | <0.001 |
| Middle occipital cortex | -32 | -92 | 0 | 6.92 |  |  |
| Inferior occipital cortex | 30 | -88 | -2 | 7.37 | 4300 | <0.001 |
| Lingual gyrus | 22 | -88 | 8 | 7.02 |  |  |
| Putamen | -16 | 12 | -8 | 5.85 | 639 | <0.001 |
|  | 20 | 6 | -4 | 5.24 | 491 | <0.001 |
|  | -28 | -12 | -8 | 3.97 | 142 | 0.008 |
| Inferior parietal cortex | -46 | -46 | 50 | 4.98 | 455 | <0.001 |
| Posterior cingulate | 2 | -38 | 28 | 4.27 | 334 | <0.001 |
| Middle occipital cortex | 30 | -66 | 30 | 4.69 | 173 | 0.003 |
| Cerebellum | -8 | -70 | -18 | 4.41 | 118 | 0.021 |

HC: Healthy controls; ANS: Absent negative symptoms; HNS: High negative symptoms; IFG: Inferior frontal gyrus; DLPFC: Dorsolateral prefrontal cortex; OFC: Orbitofrontal cortex; ACC: Anterior cingulate cortex

**Supplementary table 2.** Peaks of significant differences between groups

|  | **MNI coordinates** | | |  |  |  |
| --- | --- | --- | --- | --- | --- | --- |
| **Region** | ***x*** | ***y*** | ***z*** | ***Z*** | ***k*** | ***p*** |
| *HC > HNS* |  |  |  |  |  |  |
| IFG, *pars opercularis* | -48 | 10 | 12 | 4.65 | 518 | <0.001 |
| Middle occipital cortex | 28 | -90 | 10 | 4.52 | 142 | 0.008 |
| Medial superior frontal cortex | 4 | 70 | 20 | 4.30 | 126 | 0.015 |
| Middle temporal cortex | -58 | -48 | 2 | 4.23 | 124 | 0.017 |
|  |  |  |  |  |  |  |
| *ANS > HNS* |  |  |  |  |  |  |
| IFG, *pars orbitalis* | -50 | 22 | -4 | 4.42 | 226 | <0.001 |
| Inferior occipital cortex | -32 | -80 | -12 | 4.48 | 175 | 0.003 |
| IFG, *pars opercularis* | -32 | 12 | 30 | 4.43 | 165 | 0.004 |
